# Acute Stroke due to Vertebral Artery Dissection in Giant Cell Arteritis

**DOI:** 10.1155/2021/5518541

**Published:** 2021-06-30

**Authors:** Marlene Marte Furment, Sandra Antigua Jimenez, Sangeetha Pabolu

**Affiliations:** ^1^Internal Medicine Department, Saint Peter's University Hospital/Rutgers Robert Wood Johnson Medical School Program, New Brunswick, NJ, USA; ^2^Department of Health Sciences, Instituto Tecnologico de Santo Domingo, Santo Domingo, Dominican Republic; ^3^Rheumatology Department, Saint Peter's University Hospital/Rutgers Robert Wood Johnson Medical School Program, New Brunswick, NJ, USA

## Abstract

The diagnosis of giant cell arteritis (GCA) when presenting with atypical features such as stroke is very challenging. Only 0.17% of first-ever strokes are caused by GCA, a life-threatening condition when left untreated. Very few cases have been reported on giant cell arteritis leading to acute stroke due to vertebral artery dissection. We present a case of a 76-year-old female with no medical history who presented with sudden onset right visual loss and left hemiparesis. She had been initially treated for acute stroke and upon further workup was found to have left vertebral artery dissection. She had erythrocyte sedimentation rate (ESR) of 71 mm/h, and bilateral temporal artery biopsy was consistent with giant cell arteritis. Patient received high doses of methylprednisolone which resolved her hemiparesis, but her vision loss did not improve. Stroke in the presence of significant involvement of vertebral arteries should raise suspicion of GCA especially if classic symptoms preceded stroke event. High clinical suspicion is required to prevent delay in diagnosis and treatment.

## 1. Introduction

Giant cell arteritis (GCA) is a granulomatous inflammatory disease of medium and large vessels. It is the most frequent primary systemic vasculitis in patients over 50 years of age. It is more common in women, and genetic predisposition has been associated with this disease [1]. Classic GCA symptoms that represent cranial involvement include temporal headache, jaw and tongue claudication, trismus, diplopia, and sudden vision loss [1]. In addition, large vessel involvement of GCA has systemic manifestations such as limb claudication. We discuss a rare case of GCA presenting with left hemiparesis and right monocular visual loss from left vertebral artery dissection.

## 2. Case Presentation

A 76-year-old right-handed, Caucasian female with no significant personal history presented to the emergency room for acute onset left facial numbness, difficulty speaking, and left upper and left lower extremities weakness associated with difficulty in ambulation. She also developed painless sudden onset right eye blindness. Prior to presentation, she had 3 weeks of bilateral ear pain that was worse when eating, with no ear secretions, fevers, or hearing loss. She had visited an urgent care facility where she was treated as external otitis media with topical antibiotics and oral NSAIDs without improvement of symptoms. She subsequently developed pain over her temples and frontal scalp. Patient referred generalized myalgia and fatigue for a year which she attributed to stress without associated stiffness or pain on the shoulder or hip girdles.

On admission, her vital signs were significant for blood pressure of 169/67 mmHg, a regular heart rate of 69, and temperature of 97.3°F. Physical exam was significant for moderate dysarthria and complete right monocular visual loss with otherwise intact cranial nerves I–XIX. She had left hemiparesis with a power of 4/5 left upper and left lower extremities with brisk deep tendon reflexes and normal tone. Right-sided motor strength was normal as well as sensory exam in all four limbs. Plantar reflexes were downgoing bilaterally. Gait ataxia was present. Her National Institutes of Health Stroke (NIHS) Score was 8 points.

**Figure 1:**
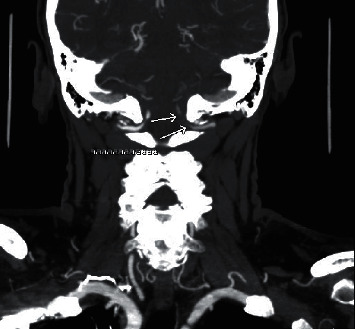
CTA showing left vertebral artery dissection in coronal plane.

**Figure 2:**
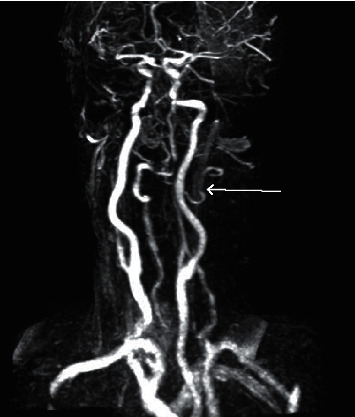
CTA showing left vertebral artery dissection.

Imaging studies were performed and were significant for a head and neck CT angiogram (CTA) that showed significant dissection of the left vertebral artery and moderate long segment stenosis involving the distal right vertebral artery (Figures 1 and 2).

Patient was started on dual antiplatelets therapy with aspirin and clopidogrel for acute stroke management as per neurology recommendations. Her erythrocyte sedimentation rate was elevated at 71 mm/h.

**Figure 3:**
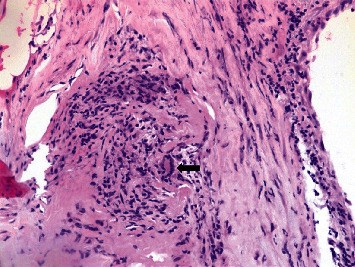
Giant cell indicated in arrow along with granulomatous changes.

Given high suspicion of giant cell arteritis, she was also treated with 3 days of pulse dose steroid followed by prednisone 1 mg/kg per day not exceeding 60 mg. She underwent bilateral temporal artery biopsy, and pathology report was consistent with giant cell arteritis (Figure 3). Her left hemiparesis resolved within 48 hours of corticosteroid initiation, but visual loss did not improve. She was discharged on slow taper of prednisone starting with 50 mg daily and aspirin 81 mg daily. Upon follow-up of 2 weeks after discharge, she was noted to have intact motor and sensory function in all four extremities, and her ESR went down to 2 mm/h, but her right monocular visual loss was not recovered.

## 3. Discussion

The typical presentation of giant cell arteritis results from vascular inflammation primarily involving external carotid branches including temporal artery and vertebral artery [1, 2]. In early phases, the symptoms can be nonspecific such as malaise, weight loss, and anorexia. Out of all, GCA classic symptoms temporal headache is the most common, and like in our case, it is usually preceded by scalp tenderness [3, 4]. Other symptoms of GCA include jaw and tongue claudication, trismus, diplopia, or sudden vision loss [5, 6].

GCA may occur concomitantly in patients with polymyalgia rheumatica presenting with symptoms of pain in shoulders and hip girdles.

The inflammatory characteristic of GCA results in partial or complete occlusion of the arterial lumen, leading to complications such as ischemic transient ischemic attack (TIA), microembolism, and most importantly, acute ischemic optic neuropathy in 15% of patients [3, 4]. Diagnosis is challenging when presenting with atypical features such as stroke as it only occurs in 2–7% of patients with GCA as per the largest population study published so far [7, 8]. An estimated 0.15% of patients with first-ever stroke will be related to GCA [9]. Stroke related to GCA is usually due to vasculitis of extracranial cerebral arteries involving vertebral or internal carotid arteries.

A relevant characteristic of stroke in patients with GCA is that up to 75% of cases will have involvement of vertebrobasilar circulation, contrasted to only 20% in the general stroke population. Typically, vertebral artery abnormalities are usually due to GCA and not atherosclerosis [10].

The chronic inflammatory process may also lead to vertebral artery dissection, as in our patient, with only two other cases documented in literature. Both cases were female patients more than 75 years old that presented similarly with headaches and bitemporal tenderness, complicated with rapid comatose state and subsequently death. In contrast to these patients, our case had unilateral vertebral artery dissection and did not have a fatal outcome. Patient characteristics are summarized in Table 1.

Temporal artery biopsy is the gold standard for diagnosis as it accounts for a high sensitivity and 100% specificity [13]. However, given emergent need of diagnosis, clinical features are the key to prevent delayed diagnosis and ultimately treatment. For this reason, American College of Rheumatology's (ACR) diagnostic criteria are used. These must include 3 out of 5 between (1) >50 years, (2) new onset headache, (3) temporal artery tenderness to palpation or decreased pulsation, (4) ESR > 50 mm/h, and (5) biopsy proven vasculitis [14].

Meeting the ACR criteria does not necessarily diagnose GCA, as other requirements need to be taken into consideration, being a positive temporal artery biopsy and ESR the main sensitivity and specificity for diagnosis [4]. These criteria have an estimated 93.5% sensitivity and a specificity of 91.2% for the diagnosis of GCA [15]. Temporal artery ultrasound and brain imaging are also emerging as the characteristic “halo sign” also helps to lead to a faster diagnosis.

Systemic glucocorticoids are the cornerstone of treatment and must be started as soon as clinical suspicion is present to decrease the risks of irreversible complications such as vision loss. Hayreh and Zimmerman determined that when glucocorticoids are started before visual involvement, it successfully brings down the risk of visual loss to 1% [16].

When no ischemic events are present, it has been accepted by expert opinion to start prednisone 1 mg/kg (maximum of 60 mg) daily which usually leads to significant symptomatic relief within 48 hours of administration. Inflammatory markers such as ESR also have similar responses.

Although there are no clinical trials or studies available to guide initial dose and tapering dose, high-dose corticosteroids are preferred up to 1000 mg of methylprednisolone daily for three days when ischemic complications such as stroke or vision loss are present [2].

Interferon gamma, interleukin-1 (IL-1), and interleukin-6 (IL-6) pathways are associated with the pathophysiology of GCA. Based on this premise, several studies have been designed to investigate new treatment options. Glucocorticoid-sparing agents such as methotrexate and the IL-6 inhibitor tocilizumab have recently emerged as an alternative for those cases where risks of prednisone use outweigh the benefits. In 2017, the GiACTA trial concluded that the IL-6 inhibitor tocilizumab combined with a course of prednisone taper was more effective than prednisone alone in leading to a sustained remission [17].

On the other hand, Mahr et al. conducted a meta-analysis that concluded that glucocorticoids combined with methotrexate decreases the risk of relapse and reduces the total amount of glucocorticoids needed to achieve clinical response [18].

Other agents such as abatacept, azathioprine, ustekinumab, and cyclophosphamide have been reported to be effective, but formal studies are still needed in literature to prove efficacy.

Moreover, low-dose aspirin in addition to corticosteroids has been reported to decrease the rate of vision loss and stroke during disease course [2].

## 4. Conclusions

First-ever stroke due to vertebral artery dissection in giant cell arteritis is extremely rare and poses a great challenge for diagnosis. For patients presenting with acute stroke with significant vertebral artery involvement, giant cell arteritis must be considered, especially if classic symptoms precede stroke presentation. Early recognition of GCA helps in providing appropriate and prompt treatment, significantly reducing the risk of irreversible complications. Glucocorticoids remain the cornerstone of management, but other agents such as tocilizumab and methotrexate are being investigated as alternatives. Certainly, more studies are required in order to better understand pathophysiology and management of these complications.

## Figures and Tables

**Table 1:** Comparison of patients' characteristics.

Age	Gender	Clinical findings	ESR	CRP	Radiological findings	Treatment	Outcome	Reference
85	F	Headache and bitemporal tenderness	40 mm/h	Not tested	Not performed	Prednisolone 60 mg/day switched to 5 mg/day after 4 months	Death 9 months after diagnosis	Sheehan et al. [11]

77	F	Right eye blurry vision, bitemporal headache, and jaw claudication	60 mm/h	5.9	CTA with V2-V3 bilateral vertebral artery dissection	Prednisolone 1 g/day	Death 6 days after diagnosis	Proft et al. [12]

## Data Availability

The data used to support the findings of this study are included within the article.

## Abbreviations

ACR: American College of Rheumatology
GCA: Giant cell arteritis
ESR: Erythrocyte sedimentation rate
CTA: Computed tomography angiogram
IL-1: Interleukin-1
IL-6: Interleukin-6
GiACTA: Giant cell arteritis actemra (GiACTA) trial.
